# The Role of the Metabolome and Non-Coding RNA on Pheochromocytomas and Paragangliomas: An Update

**DOI:** 10.3390/metabo12020131

**Published:** 2022-02-01

**Authors:** Bruno Bouça, Paula Bogalho, Manfredi Rizzo, José Silva-Nunes

**Affiliations:** 1 Department of Endocrinology, Diabetes and Metabolism, Centro Hospitalar Universitário Lisboa Central, 1069-166 Lisbon, Portugal; pbogalho@gmail.com (P.B.); silva.nunes@nms.unl.pt (J.S.-N.); 2 Faculdade de Ciencias Medicas, Nova Medical School, Universidade Nova de Lisboa, 1169-056 Lisbon, Portugal; 3 Department of Health Promotion, Mother and Child Care, Internal Medicine and Medical Specialties, University of Palermo, 90100 Palermo, Italy; manfredi.rizzo@unipa.it; 4 Health and Technology Research Center (H&TRC), Escola Superior de Tecnologia da Saude de Lisboa, 1990-096 Lisbon, Portugal

**Keywords:** pheochromocytoma, paraganglioma, metabolome, microRNA, epigenetics

## Abstract

Pheochromocytoma and paragangliomas (PPGL) are rare neuroendocrine tumors. In some patients they exhibit malignant behavior characterized by the presence of metastases, limiting treatment options and survival rates. Therapeutic options are limited to surgery, localized radiotherapy, and a few systemic therapies. However, in several recent studies, non-coding RNA molecules are gaining increasing attention as markers of malignancy for PPGL. The understanding of PPGL development molecular mechanisms has improved in the last years, with some of the epigenetic regulatory mechanisms such as DNA and histones methylation, being better understood than RNA-based mechanisms. Metabolome deregulation in PPGL, with increased synthesis of molecules that facilitated tumor growth, results from the activation of hypoxia signaling pathways, affecting tumorigenesis. In addition, the assessment of these metabolites can be useful for the management of these tumors. This review summarizes recent discoveries linking metabolome and non-coding RNA to PPGL and their relevance for diagnosis and therapeutics.

## 1. Introduction

Pheochromocytoma and paragangliomas (PPGL) are rare neuroendocrine tumors. They originate from cells of the embryonic neural crest, which develop into the sympathetic and parasympathetic ganglia, derived from the ectoderm. Nearly 80% of the tumors arise from the adrenal gland, while approximately 15–20% originate from extra-adrenal locations [1].

These tumors are associated with excessive secretion of catecholamines and generally exhibit slow-growing, most of them do not metastasize and are curable with surgery. Because of molecular techniques expertise, it’s now known that at least 30–40% occur in the context of hereditary disease. The most common genetic abnormalities that have been reported are mutations in the succinate dehydrogenase genes (*SDHA*, *SDHB*, *SDHC*, and *SDHD*), accounting for up to half of all germline alterations in pheochromocytomas and paragangliomas. Von Hippel-Lindau syndrome (*VHL*), multiple endocrine neoplasia type 2 (*RET*), and neurofibromatosis type 1 (*NF1*) are the other most frequent germline mutations [2]. However, in some patients (10–30%, according to different studies), PPGL exhibits malignant behavior characterized by the presence of metastases, limiting treatment options and survival rates; only 60% of patients will survive five years after initial diagnosis [3]. The understanding of metastatic PPGL is scant, and the management of these tumors represents a striking challenge. The natural course of the disease is highly heterogeneous, depending on the presence of predictors of rapid disease progression and death-older age at primary tumor diagnosis, larger primary tumor size (>5 cm), failure to undergo surgical resection of the primary tumor, and presence of synchronous metastases. While head and neck PPGL show more indolent courses and longer survival rates, other extra-adrenal tumors have been associated with a higher risk for metastatic spread [4]. Therefore, overall survival, progression-free survival, and clinical outcome are difficult to predict for an individual patient.

Therapeutic options that can improve prognosis are limited to surgery, localized radiotherapy, and a few systemic therapies, such as chemotherapy and metaiodobenzylguanidine iodine-131 (I-131-MIBG). However, very often the outcome results in incomplete responses that commonly last for a short period of time [3]. 

A further challenge comes from the fact that there is a 15–20% chance of recurrence of PPGLs over a 10-year period and a 20% malignancy rate that makes long-term surveillance mandatory [1]. For this reason, knowledge of the genetic and molecular pathways is of paramount importance for understanding tumorigenesis and helping with the development of targeted therapies. 

The understanding of the molecular mechanisms in PPGL development has improved in recent years, but the factors influencing metastasis development are still largely unknown. Predefined algorithms such as the Pheochromocytoma of the Adrenal Gland Scaled Score (PASS) and the Grading System for Adrenal Pheochromocytoma and Paraganglioma (GAPP) have been suggested to predict metastatic behavior of these tumors, showing a good negative predictive value [5,6]. Unfortunately, inter-observer variability and lack of accuracy make their clinical use difficult; therefore, the understanding of tumorigenesis pathways is paramount to identifying predictive biomarkers that may assist therapeutic decision-making. The metabolome is made up of molecules produced by cellular enzymatic activity, representing the product of the full interaction between the genome, epigenetics, and environmental factors [7]. The metabolic changes identified in PPGL reflect the activation of hypoxia pathways, with similarity in PPGL with different genetic origins. The assessment of the metabolome characteristics has allowed the validation of enzymatic defects in some PPGL, such as the role of fumarate hydratase (*FH*), isocitrate dehydrogenase (*IDH*), malate dehydrogenase (*MDH2*), and aspartate transaminase (*GOT2*) [8]. RNA-based mechanisms were also found to have a profound role in physiological processes such as immunity, metabolism, cell apoptosis, differentiation, proliferation, and control of angiogenesis [9]. 

**Figure 1:**
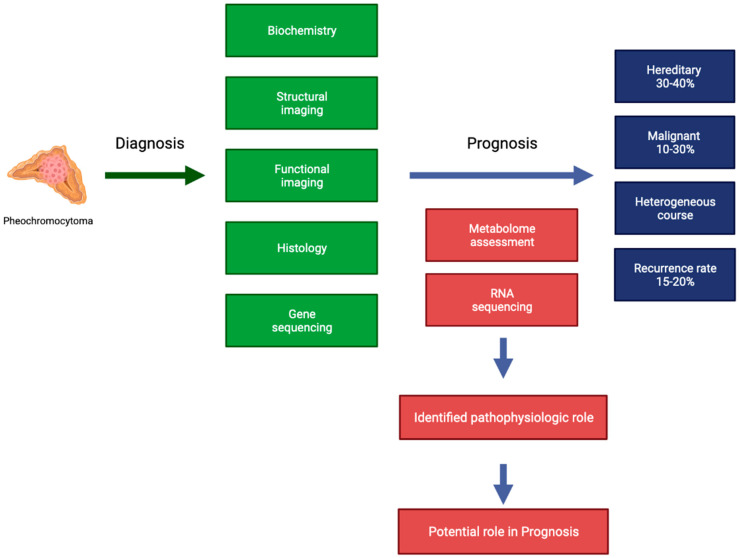
Potential role of metabolome assessment and RNA sequencing in PPGL management.

Given the difficulties in PPGL management, especially the lack of markers of malignancy, non-coding RNA (ncRNA) molecules are gaining increasing attention, similarly to other neoplasms [10,11,12]. Recently, the pattern of expression of ncRNAs, including microRNAs (miRNAs) and long non-coding RNAs (lncRNAs), gained attention in metastatic PPGLs. This review aims to discuss some of the genetic mechanisms of PPGL development and novel insights about epigenetics and metabolome (Figure 1).

## 2. Metabolome

**Figure 2:**
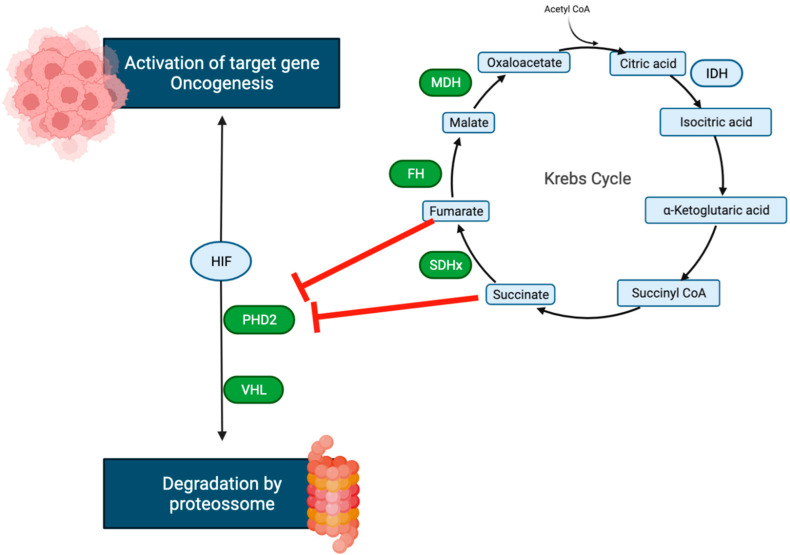
Prolyl hidroxylase (PHD) hydroxylates two proline residues in HIF subunits under healthy conditions, allowing the von Hipple-Lindau protein (*VHL*) to recognize them. *VHL* is part of a ubiquitination protein complex. The activity of PHD is dependent on oxygen and oxoglutarate. When oxygen levels fall below physiological levels, PHD activity is inhibited, resulting in *VHL* dissociation from HIF and HIF stabilization. HIF is then transported to the nucleus, where it binds to HIF and promotes transcription of target genes and oncogenesis. When present at high concentrations, succinate, like fumarate, structurally mimics oxoglutarate and inhibits PHD and activates transcription of target genes by binding to hypoxia-responsive elements (HRE) in their promoter regions. When present in high amounts, succinate, like fumarate, structurally mimics 2-OG and inhibits PHDs (product inhibition), as shown in tumor cells.

**Figure 3:**
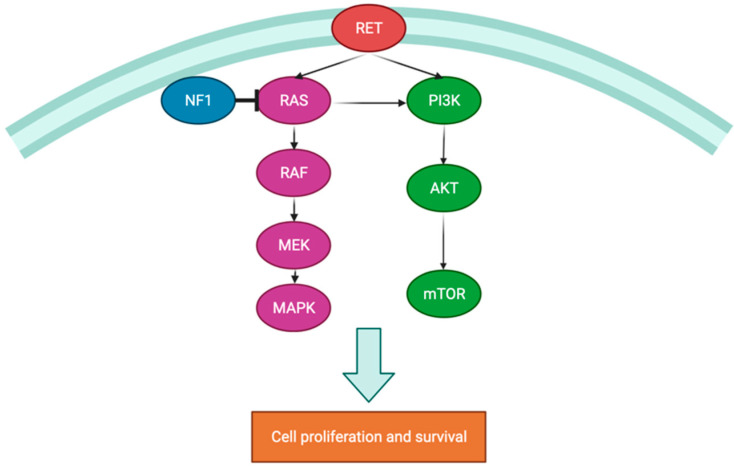
Schematic review of kinase signaling pathway in the pathogenesis of PPGLs (cluster 2).

The metabolome is defined as the set of small molecules present in the cell and in its proximal environment, essential to its functions and maintenance, which reflect the result of biochemical reactions and determine its phenotype. It is influenced by genetic, environmental, and epigenetic factors, which may correspond to an indirect way of diagnosing cellular dysfunction [8]. Metabolome deregulation in PPGL results from the activation of hypoxia signaling pathways. Such an increased synthesis of molecules (D-2-hydroxyglutarate, fumarate, succinate) can influence tumor growth, facilitating tumorigenesis. In PPGL, this occurs directly through mutations involved in the regulation of hypoxia response (cluster 1) or indirectly through mutations in the mTOR and *phosphatidylinositol 3*-*kinase* (*PI3K*) pathways (cluster 2) (Figure 2 and Figure 3) [13,14]. PPGL may express defects in Krebs cycle enzymes, the most common being at the level of succinate dehydrogenase, resulting in an increase in succinate and a reduction in fumarate levels. More rarely, defects in fumarate hydratase, malate dehydrogenase, or isocitrate may also occur [14,15]. These alterations in the metabolome are congruent with the enzymatic defects identified in some types of PPGL, allowing the suspicion of these genetic variants. In PPGL with succinate dehydrogenase (*SDH*) complex mutations, there is an accumulation of several metabolites such as succinate, methionine, and glutamine and decreased fumarate, glutamate, and isocitrate levels [15,16,17]. In addition, measurement of these metabolites can be useful for the diagnosis of variants that have not been identified so far, with possible implications for the clinical approach. As an example, the succinate/fumarate ratio may play an important role in determining the risk for metastasis development and response to therapy; that ratio would be a good indicator for distinguishing between *SDH* and non-*SDH* mutations [17,18,19].

In line with these facts, high levels of succinate and fumarate have been shown to be responsible for activating hypoxia signaling pathways and tumorigenic mechanisms such as hypermethylation [20,21]. The decrease in intermediate metabolites of the Krebs’ cycle also plays a role in the epigenetic and metabolomic alterations of PPGL, and it has been shown that the reduction in citrate levels is correlated with tumor local invasiveness and metastasis development [22]. It is believed that the differences in the synthesis and secretion of catecholamines in different types of PPGL may be explained by adjustments in these metabolic pathways. For instance, levels of ascorbate, a cofactor in the conversion of dopamine to noradrenaline, show a direct correlation with the concentration of catecholamines. Thus, the assessment of the metabolites mentioned above is of particular importance in patients with PPGL without increased levels of catecholamines/metanephrines, allowing the differentiation of *SDH* and non-*SDH* tumors [16,23].

## 3. RNA

RNAs play different roles in cell regulation processes, such as biochemical reactions similar to those performed by enzymes; additionally, it is involved in complex regulatory functions that are related to the pathophysiology of various diseases [24,25]. Recently, the role of RNA mechanisms in epigenetic regulation and their implication in the pathogenesis of several tumors, namely long non-coding, miRNA, and Circular RNA (circRNA), has been highlighted. These molecules have also been proposed as potential diagnostic and therapeutic targets [24,25,26].

The main mechanisms, identified so far, are modifications in gene expression through chromatin remodeling, transcriptional, and post-transcriptional regulation [27,28].

### 3.1. MicroRNA (miRNA)

miRNAs are small, single-stranded, and evolutionary conserved non-coding RNA molecules with 19−25 nucleotides, with specific and variable expression in certain organs/tissues. It is estimated that miRNA regulates at least 30% of protein-coding genes and accounts for 1−5% of the human genome. They exhibit a behavior similar to that of transcription factors but act at the level of the target mRNA, by degradation or by blockage of cytoplasmatic translation. Thus, the same type of miRNA may have a tumor suppressor or activator role depending on the tissue in which it is expressed, which makes its role even more complex [29,30,31].

Some of the identified biological functions for miRNAs include: (a) cell cycle regulation, (b) cell proliferation and differentiation, (c) apoptosis, (d) hormonal secretion, (e) immune regulation, and (f) control of angiogenesis. Furthermore, the role of miRNAs in tumorigenesis and vascular or autoimmune diseases has been extensively studied. An association between the expression of some miRNA and different pathologies has been assumed [32].

MicroRNAs have been identified in several neoplasms—breast, lung, and gastric cancer—showing great relevance in determining tumor behavior, especially when the histological analysis is unclear [33]. Table 1 describes the different types of microRNA identified in PPGL and their possible diagnostic/therapeutic utility.

Currently, we know that the malignant behavior of PPGL cannot be determined by histological analysis; we also know that there are no unquestionable biological markers for that purpose [34,35]. If we demonstrate that the expression of different microRNAs can be specific for each one of the clusters, we may assume a future potential role for these molecules in PPGL management.

### 3.2. Long Non-Coding RNAs (lncRNA)

lncRNAs are made up of more than 200 nucleotides and are non-coding RNAs, whose functions are not well understood but are believed to play a key role in gene regulation and in the interaction of DNA, RNA, and proteins. Like microRNAs, lncRNAs also seem to be specific to each tissue and associated pathology, and their function depends on their location in different cell compartments, assuming their role as biomarkers [25,42]. Thus, at different stages of metabolism and cell interaction, lncRNA can play major roles, namely conditioning the tumor metabolome that is favorable to its development.

Recently, some lncRNA (DGCR9, FENDRR, HIF1A-AS2, MIR210HG, and BC063866) were associated with metastatic PPGL; lncRNA BC063866 showed a particularly strong association with *SDH* mutations [25,42]. Furthermore, through bioinformatics analysis, it has been shown that lncRNA BSNAS2 could interact with microRNA mi195 and be associated with poor prognosis. The decreased expression of lncRNA C9orf147 and PTPRJ were associated with a better prognosis [43].

Large-scale studies will be essential to identify and validate these lncRNA as biomarkers and their potential use in PPGL management.

### 3.3. Circular RNA (circRNA)

circRNAs are formed by backsplicing from the intron-containing pre-mRNA. Initially considered redundant products of gene transcription, they are highly conserved and stable non-coding RNAs, of nuclear or cytoplasmic localization, that participate in the regulation of several biological processes [44]. There are six mechanisms through which circRNA interfere with gene expression: (a) stimulation of transcription through the action on RNA polymerase II, (b) attenuation of splicing mechanisms through mRNA, (c) inhibition of gene translation, (d) encoding proteins involved in the gene transcription process, (e) formation of protein complexes that alter enzymatic activity, and (f) miRNA sequestration, forming miRNA sponges [41,45].

Considering all the mechanisms by which circRNA can interfere in gene expression, it is believed that circRNA may play a crucial role in tumorigenesis. Evidence for this is the fact that it has been shown that there is an alteration in the expression of these RNAs in several tumors, such as the bladder, kidney, breast, and colon [46]. Due to their stability, superior when compared to other types of RNA, and specific expression in different tissues, these RNAs are pointed out as a possible source of new biomarkers for screening and therapeutic targets [47].

There is only one study that investigated the relevance of circular RNA in PPGL, suggesting that its role is through histone methylation. However, due to the low number of patients, the characteristics of the study, and the use of bioinformatics predictions, these results lack experimental validation [48].

## 4. Conclusions

More than 40% of patients with PPGL have germline mutations, justifying that all individuals with the disease should undergo genetic testing. The evolution in diagnosis has been notorious, not only through the greater sensitivity and specificity of biochemical methods but also through the improvement of imaging methods that enable the location of lesions, even in asymptomatic patients (incidentalomas). Presently, no histological, molecular, or genetic characteristics can differentiate, with absolute certainty, between the benign and malignant behavior of PPGL. The treatment of malignant PPGL also remains a challenge, as available therapies are still not curative, and many tumors are resistant to chemotherapy or radiotherapy. Research in metabolomics and genomics of these tumors can allow the discovery of susceptible mechanisms of action for new therapies. The form of the disease associated with *SDHB* germline mutation is of greatest interest, as it is present in approximately 30% of malignant PPGL. The growing number of studies demonstrating the role of miRNA and other non-coding RNAs in tumorigenesis and their role in the pathophysiology of PPGL will probably justify their clinical use in the future. Likewise, the study of the metabolome associated with specific tumors, such as the case of pheochromocytoma and paraganglioma, may become routine for diagnosis of the specific molecular changes associated with these neoplasms, and for optimization of the treatment. Additionally, the exponential increase in phase II trials for therapeutic agents acting on the natural history of PPGL allows us to optimistically expect advances in the near future.

## Figures and Tables

**Table 1:** miRNA with altered expression in PPGL and their possible role in diagnosis and therapeutics.

Micro RNA	Expression Alteration and Possible Role
miRNA 15 [36] and miRNA 16 [36]	Underexpression in metastatic pheochromocytoma Tumor suppressor–promotes cell death
miRNA 21-3p [37]	Associated with higher sensitivity to rapamycin
miRNA 96 [38]	Overexpression in *SDHB* type
miRNA 101 [39]	Overexpression in *SDHB* type and metastatic PPGL
miRNA 133 [33,38]	Overexpression in *VHL* type
miRNA 137 [33]	Overexpression in most PPGL
miRNA 139-3p [33,38]	Overexpression in *VHL* type
miRNA 148-3p [40]	Associated with good prognosis
miRNA 183 [39]	Overexpression in *SDHB* type
miRNA 193 and miRNA 195 [41]	Downregulated in PPGL
miRNA 210 [9]	Overexpression in *SDH* and *VHL* types Possibly associated with more aggressive disease
miRNA 338-3p [40]	Associated with good prognosis
miRNA 375 [41]	Overexpression in most PPGL
miRNA 382 [38]	Overexpression in *VHL*, *SDHB*, *SDHD*, and RET mutated PPGL
miRNA 483-5p [36,39]	Overexpression in metastatic PPGL Associated with worse survival rate
miRNA 488 [38]	Overexpression in MEN2 associated PPGL
miRNA 497 and miRNA 508 [41]	Downregulated in PPGL
miRNA 541 and miRNA 765 [33]	Overexpression in *VHL* type
miRNA 885 [33]	Overexpression in MEN2 associated PPGL
miRNA 1225-3p [33]	Overexpression in recurrent PPGL
